# Bacterial ClpP Protease Is a Potential Target for Methyl Gallate

**DOI:** 10.3389/fmicb.2020.598692

**Published:** 2021-02-04

**Authors:** Dehong Zheng, Yanan Xu, Gaoqing Yuan, Xiaogang Wu, Qiqin Li

**Affiliations:** ^1^State Key Laboratory for Conservation and Utilization of Subtropical Agro-Bioresources, College of Agriculture, Guangxi University, Nanning, China; ^2^Pharmaceutical College, Guangxi Medical University, Nanning, China

**Keywords:** methyl gallate, protease ClpP, drug target, transposon sequencing, *Ralstonia solanacearum*

## Abstract

Methyl gallate (MG) is an effective microbicide with great potential application in the integrated management of plant diseases and an important potential drug for clinical application. However, its target remains unknown. This study conducted a transposon sequencing (Tn-seq) under MG treatment in plant pathogenic bacterium *Ralstonia solanacearum*. Tn-seq identified that the mutation of caseinolytic protease proteolytic subunit gene *clpP* significantly increased the resistance of *R. solanacearum* to MG, which was validated by the in-frame gene deletion. iTRAQ (isobaric tags for relative and absolute quantitation) proteomics analysis revealed that chemotaxis and flagella associated proteins were the major substrates degraded by ClpP under the tested condition. Moreover, sulfur metabolism-associated proteins were potential substrates of ClpP and were upregulated by MG treatment in wild-type *R. solanacearum* but not in *clpP* mutant. Furthermore, molecular docking confirmed the possible interaction between MG and ClpP. Collectively, this study revealed that MG might target bacterial ClpP, inhibit the activity of ClpP, and consequently disturb bacterial proteostasis, providing a theoretical basis for the application of MG.

## Introduction

Methyl gallate (MG) (methyl 3,4,5-trihydroxybenzoate) is a well-studied polyphenolic compound, which is isolated from many plants, such as *Toxicodendron sylvestre* (Yuan et al., 2012), *Tamarix nilotica* (Orabi et al., 2020), baru (*Dipteryx alata* Vog.) (Oliveira-Alves et al., 2020), and walnuts (*Juglans regia* L.) (Zhang et al., 2020). MG is an effective microbicide, which shows great potential application in the integrated management of plant diseases, such as tomato bacterial wilt caused by *Ralstonia solanacearum* (Yuan et al., 2012) and rice blast caused by *Magnaporthe grisea* (Ahn et al., 2005). Moreover, MG is an important potential drug for clinical application. MG effectively inhibits the adhesion, invasion, and intracellular survival of *Salmonella typhimurium* (Birhanu et al., 2018). MG also inhibits oral bacterial growth and the formation of *Streptococcus mutans* biofilm (Kang et al., 2008). The activity of antileishmanials (Noleto Dias et al., 2020) and the inhibition of colitis (Anzoise et al., 2018) and osteoclast (Baek et al., 2017) have been reported. MG is a potent and highly specific inhibitor of herpes simplex virus *in vitro* (Kane et al., 1988). Recent virtual screening has shown that MG is a potential ligand binding the NSP10/NSP16 methyltransferase of coronavirus disease 2019 (COVID-19) (Maurya et al., 2020). However, the molecular target for MG remains unknown.

ClpP, a caseinolytic protease proteolytic subunit, is a highly conserved self-compartmentalizing processive serine protease playing an important role in proteostasis of prokaryotic cells and eukaryotic organelles (Moreno-Cinos et al., 2019). ClpP of *Escherichia coli* and *Bacillus subtilis* degrades proteins involved in transcription regulation, metabolic enzymes, starvation and oxidative stress responses, and DNA damage repair (Flynn et al., 2003; Kock et al., 2004). In addition, the important roles of ClpP in bacterial pathogenesis are widely reported. ClpP regulates the expression of genes in *Salmonella* pathogenicity island 1 and is essential for survival within the peritoneal macrophages (Leung and Finlay, 1991; Knudsen et al., 2013). ClpP is involved in the level of hemolytic factor α-hemolysin, heme-iron extracting Isd proteins, and consequently the pathogenesis in *Staphylococcus aureus* (Frees et al., 2003; Farrand et al., 2013). In the human mitochondria, ClpP regulates the homeostasis of proteins involved in the cellular metabolic pathways such as the electron transport chain, and the expression of ClpP is related to carcinomas, infertility, and sensorineural deafness of Perrault syndrome (Moreno-Cinos et al., 2019).

Given its crucial roles in bacterial pathogenesis and human diseases, ClpP is an attractive drug target. Using activity-based protein profiling, several trans-β-lactone compounds are identified as ClpP inhibitors (Evans and Cravatt, 2006). An optimized β-lactone U1 inhibits ClpP from *S. aureus*, *Listeria monocytogenes*, *Plasmodium falciparum*, and *Mycobacterium tuberculosis* (Bhandari et al., 2018). Other identified ClpP inhibitors include phenyl esters (Hackl et al., 2015), heterocycles, pyrazolopyridine, and 2-(thiophen-2-yl)oxazole moieties (Pahl et al., 2015). Rather than inhibiting ClpP activity, the acyldepsipeptide (ADEP) family compounds dysregulate (or activate) the function of the ClpP protease, and some optimized ADEPs exhibit potent and broad-range bactericidal activity (Alexopoulos et al., 2012).

Transposon sequencing (Tn-seq) is a high-throughput technology used in identifying essential and conditionally essential genes on a genome-wide scale (van Opijnen et al., 2009). Tn-seq can be applied for the identification of genes required for resistance to bactericides (Lai et al., 2017; Vitale et al., 2020) and potential antimicrobial target genes. For instance, the potential targets of multiple antibiotics were identified in the *Acinetobacter baumannii via* Tn-seq (Geisinger et al., 2020). *R. solanacearum* is probably the most destructive plant pathogenic bacterium worldwide (Mansfield et al., 2012). Many plant-derived compounds including hydroxycoumarins (Yang et al., 2018), protocatechualdehyde (Li et al., 2016), and MG (Yuan et al., 2012) have shown potential for the effective biological control of plant bacterial wilt caused by *R. solanacearum*.

To identify the target of MG, we conducted a Tn-seq analysis in *R. solanacearum* under the treatment of MG. The mutation of *clpP* resulted in the resistance to MG, which was validated by in-frame gene deletion. The substrates of ClpP were then identified by iTRAQ (isobaric tags for relative and absolute quantitation) proteomics analysis. Proteins involved in sulfur metabolism, one category of ClpP substrates, were upregulated by MG in *R. solanacearum* wild-type strain but not in *clpP* mutant. These findings indicate that MG may bind ClpP, which is confirmed by molecular docking, inhibit the protease activity of ClpP, and consequently disturb bacterial proteostasis.

## Materials and Methods

### Bacterial Growth Conditions

All *R. solanacearum* strains were cultured in BG medium (10 g/L bacto peptone, 1 g/L casamino acids, 1 g/L yeast extract, and 5 g/L glucose) or on BG agar medium at 28°C, except for particular circumstances. *E. coli* strains were grown in LB liquid medium or on LB agar medium at 37°C. Kanamycin was added at the concentration of 25 μg/ml when needed.

### Transposon Sequencing Under Methyl Gallate Treatment

The near-saturated transposon insertion library of *R. solanacearum* GMI1000 (Su et al., 2020) preserved at −80°C was adjusted to OD600 ∼0.8 in BG medium and reactivated at 28°C for 1 h (van Opijnen et al., 2014). The reactivated transposon insertion library was then cultured in BG medium added with 25 μg/ml of MG or solvent to the logarithmic growth phase (OD600 ∼0.8). The MG-treated and untreated transposon insertion library samples were subjected to Illumina sequencing library construction, Illumina sequencing, and raw data preprocessing as previously described (Su et al., 2020). The reads mapped to the reference genome (GCA_000009125.1) were subjected to sample correlation coefficient computation *via* multiBamSummary of deepTools. The correlation coefficient was visualized *via* plotCorrelation of deepTools on the basis of the output of multiBamSummary (Ramirez et al., 2016). The reads mapped were finally analyzed by TSAS, a Tn-seq analysis software (Burger et al., 2017), using the two-sample analysis option. The preprocessed reads for mapping and the wig files from TSAS were deposited in Figshare (10.6084/m9.figshare.12869930). The analysis results were outputted in Supplementary Table S1. Ratio_reads (MG/CK) > 2 or <0.5 with the adjusted *P* value (proportions_reads) ≤ 0.01 were respectively set as the threshold value to identify the potential target or resistance genes for MG. The transposon insertion distribution in *clpP* was visualized by Integrative Genomics Viewer (Thorvaldsdóttir et al., 2012).

### Gene Deletion in *R. solanacearum*

Genes in *R. solanacearum* were deleted through consecutive homologous recombination. In brief, two flanking DNA fragments (500–800 bp) of target genes were amplified using the primers listed in Supplementary Table S2 and cloned to the suicide plasmid pK18mobsacB by three appropriate restriction sites. The resulting recombinant plasmid verified by Sanger sequencing was then transferred into *R. solanacearum* using electroporation, generating the recombined mutant. The resulting strains were subsequently cultured on the modified BG medium in which the glucose was replaced by 10% sucrose to obtain the second crossover recombined strains. The gene-deleted mutant was screened from the strains losing kanamycin resistance by polymerase chain reaction (PCR).

### The Sensitivity of *R. solanacearum* to Methyl Gallate

In assaying the sensitivity to MG, *R. solanacearum* strains were cultured to the logarithmic growth phase and adjusted to the same concentration. The adjusted bacterial cultures after gradient dilution were grown on BG agar medium with or without 25 μg/ml of MG. The growth of *R. solanacearum* strains was observed and photographed at certain times. The adjusted bacterial cultures were inoculated in BG liquid medium with or without 25 μg/ml of MG with three biological repeats, and the growth of each strain was measured by OD600 at certain times after inoculation. Growth differences between mutants and the wild-type strain GMI1000 with or without MG treatments at 23 and 31 h post inoculation were statistically analyzed by analysis of variance (ANOVA).

### iTRAQ Proteomics Analysis

*Ralstonia solanacearum* wild-type strain GMI1000 and mutant Δ*clpP* were cultured in BG liquid medium with or without 25 μg/ml of MG to the logarithmic growth phase (OD600 ∼0.8), namely, WT, WT_MG, Δ*clpP*, and Δ*clpP*_MG. Each strain and treatment were performed with two biological repeats. iTRAQ proteomics analysis was subsequently performed at Novogene (Beijing, China). In brief, total proteins were extracted by grounding in liquid nitrogen and lysed with lysis buffer containing 100 mM NH_4_HCO_3_ (pH 8), 8 M urea, and 0.2% sodium dodecyl sulfate (SDS). The extracts were then reduced with dithiothreitol (DTT) and alkylated with sufficient iodoacetamide, followed by precipitation with acetone. Proteins were digested with trypsin after quantification. Purified peptides were labeled with iTRAQ labeling reagent and fractionated by high-performance liquid chromatography (HPLC). The separated peptides were analyzed by a Q Exactive HF-X mass spectrometer (Thermo Fisher) and searched separately against the proteome of *R. solanacearum* GMI1000 (GCA_000009125.1) by Proteome Discoverer 2.2. Identified proteins were quantified and compared between certain *R. solanacearum* strains and treatments. The proteins with >1.5-fold expression difference between the experimental and control groups and with *P* < 0.05 statistically analyzed by *T*-test were defined as differentially expressed proteins. The mass spectrometry proteomics data have been deposited to the ProteomeXchange Consortium *via* the PRIDE (Perez-Riverol et al., 2019) partner repository with the dataset identifier PXD021102. To analyze the pathway, *R. solanacearum* GMI1000 proteins were mapped to the Kyoto Encyclopedia of Genes and Genomes (KEGG) database using the interproscan program (Jones et al., 2014). The hypergeometric test was then used to analyze the KEGG enrichment of differentially expressed proteins.

### Molecular Docking

The 3D ClpP structure (PDB ID: 1YG6) (Bewley et al., 2006) was obtained from protein data bank (Burley et al., 2018). The structures of MG and other ligand compounds were obtained from ZINC, a free database of commercially available compounds for virtual screening (Sterling and Irwin, 2015). Chain A of the heptameric ClpP structure was extracted for docking after removing the water using PyMOL. Chain A of ClpP and the compounds were docked and analyzed using SwissDock (Grosdidier et al., 2011). The best docking pose between MG and ClpP and the structures of ClpP binding with (4S)-2-methylpentane-2,4-diol (MPD) (PDB ID: 1YG6) and benzyloxycarbonyl-leucyltyrosine chloromethyl ketone (Z-LY-CMK) (PDB ID: 2FZS) were visualized using UCSF Chimera (Pettersen et al., 2004).

## Results

### Transposon Sequencing Analysis of Gene Essentiality for Methyl Gallate

**FIGURE 1:**
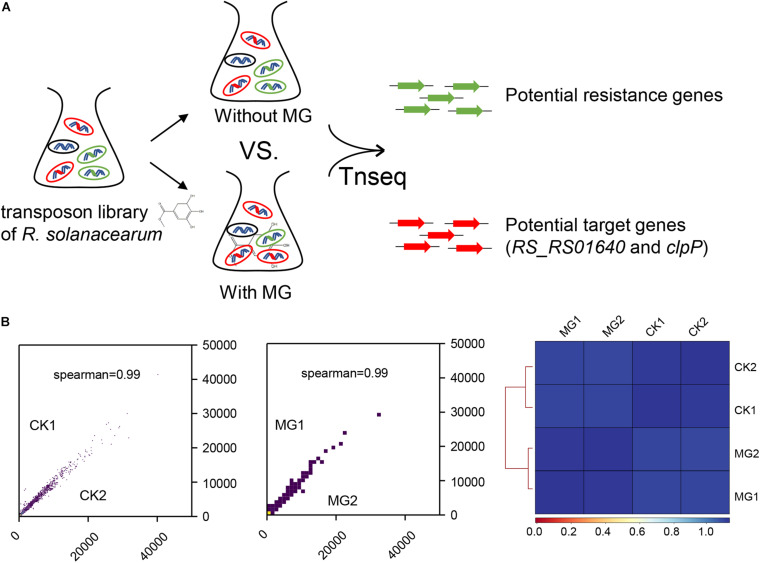
Transposon sequencing (Tn-seq) analysis of gene essentiality for methyl gallate (MG). **(A)** Schematic diagram of Tn-seq for the identification of resistance genes and target genes for MG. **(B)** Coefficient of reads coverages for Tn-seq biological replicates (scatter plot) and for all Tn-seq samples (heatmap).

To identify the target of MG, we conducted Tn-seq to analyze the gene essentiality for MG using the near-saturated transposon insertion library of *R. solanacearum* GMI1000 (Su et al., 2020). As shown in Figure 1A, *R. solanacearum* transposon insertion libraries were cultured in BG medium with or without MG to the same concentration in the logarithmic growth phase. Both treatments were performed with two biological repeats, namely, MG1/MG2 and CK1/CK2, respectively. The MG-treated and untreated transposon insertion libraries were subjected to Illumina sequencing and bioinformatics analysis to identify the relative abundance of each insertion mutant.

As shown in Figure 1B, the biological repeats were highly correlated with each other, indicating the reliability and repeatability of this analysis. A total of 148,290 unique transposon insertion hits were mapped within genes with 12,654,331 sequenced reads for CK1, and 145,572 unique hits with 12,071,932 reads were mapped within genes for CK2. MG1 and MG2 contained 149,428 unique hits with 12,906,565 reads and 147,848 unique hits with 12,682,652 reads, respectively. The transposon insertion within MG target genes would increase the relative abundance of *R. solanacearum* under MG treatment. Setting the threshold value as ratio_reads (MG/CK) > 2 or <0.5 with the adjusted *P-*value (proportions_reads) ≤ 0.01, only two genes were identified as potential target genes, and no gene was identified as a potential resistance gene for MG (Supplementary Table S1 and Table 1).

**TABLE 1:** Potential target genes for MG identified by Tn-seq.

Gene ID	Ave. Unique hits (MG)	Ave. Unique hits (CK)	Ave. Weighted reads (MG)	Ave. Weighted reads (CK)	Ratio_reads (MG/CK)	Adj. *P* (proportions_reads)
*RS_RS08645* (*clpP*)	15	7	127.5	60.5	2.1	9.94E-04
*RS_RS01640*	21	8.5	168.5	64	2.6	7.05E-08

*MG, methyl gallate; Tn-seq, transposon sequencing.*

### ClpP Is Necessary for the Antibacterial Activity of Methyl Gallate Against *R. solanacearum*

**FIGURE 2:**
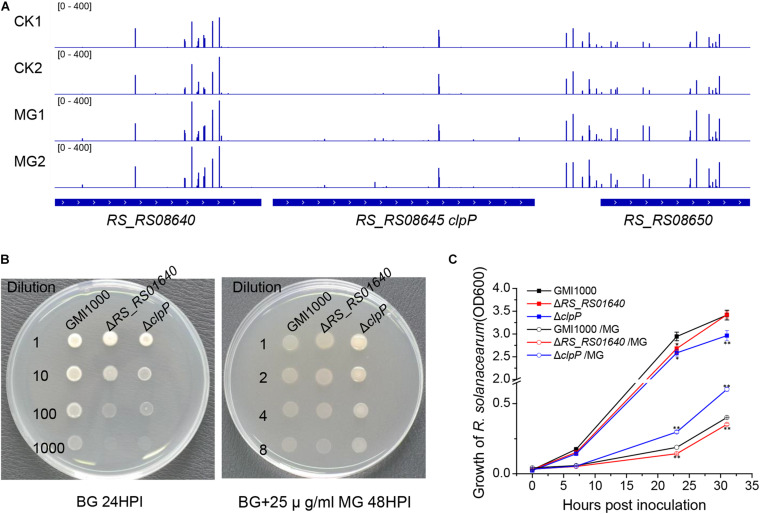
ClpP is necessary for the antibacterial activity of methyl gallate (MG) against *R. solanacearum*. **(A)** Transposon insertion distribution of *clpP* and its flanking sequence. **(B)** Gradient diluted bacterial cultures were inoculated on BG agar medium with or without 25 μg/ml of MG. The growth of *R. solanacearum* strains was observed and photographed at certain times. **(C)** Growth of *R. solanacearum* strains cultured in BG liquid medium with or with 25 μg/ml of MG. Growth differences between mutants and the wild-type strain GMI1000 with or without treatments at 23 and 31 h post inoculation are marked with asterisks, indicating *P* < 0.01 (**) or *P* < 0.05 (*), statistically analyzed by analysis of variance (ANOVA).

As shown in Table 1 and Figure 2A, 15 unique transposon insertion hits within *RS_RS08645* (*clpP*) were mapped by 127.5 reads for MG-treated samples. Transposon-inserted mutants of *clpP* were sequenced 2.1 times under MG treatment, indicating that the relative abundance of *clpP* mutants was increased by 2.1 times in MG-treated samples compared with that of the untreated samples. Similarly, transposon insertion of membrane protein gene *RS_RS01640* improved the relative abundance of *R. solanacearum* under MG treatment by 2.6 times.

*clpP* and *RS_RS01640* were deleted in-frame to verify the result of Tn-seq. As shown in Figures 2B,C, *clpP*-deleted mutant (Δ*clpP*) and *RS_RS01640*-deleted mutant (Δ*RS_RS01640*) showed attenuated growth on BG agar medium and in BG medium, indicating that ClpP and RS_RS01640 are critical for bacterial survival. Consistent with the result of Tn-seq, Δ*clpP* grew better than the wild-type strain GMI1000 under the MG treatment, indicating that ClpP is important for the full bactericidal activity of MG in *R. solanacearum*.

### Identification of ClpP Substrates by iTRAQ

**FIGURE 3:**
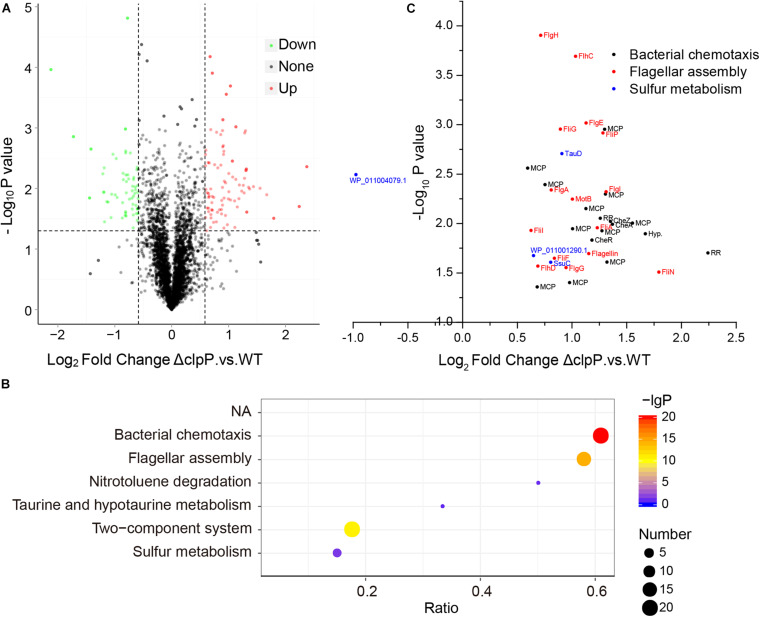
Possible substrates of ClpP identified by iTRAQ (isobaric tags for relative and absolute quantitation). **(A)** Relative expression of proteins identified in Δ*clpP* compared with the wild-type (WT) strain GMI1000. The proteins with >1.5-fold expression difference between Δ*clpP* and WT and with *P* < 0.05 were defined as differentially expressed proteins. **(B)** Kyoto Encyclopedia of Genes and Genomes (KEGG) enrichment of differentially expressed proteins of Δ*clpP* versus WT. The number of differentially expressed proteins is indicated by the size of the bubbles. *P*-values of enrichment are indicated by the color of bubbles. The ratio represents the number of proteins identified in this pathway divided by the number of differentially expressed proteins mapped to this pathway. The top six over enriched KEGG pathways were shown. **(C)** Relative expression of differentially expressed proteins mapped to bacterial chemotaxis, flagellar assembly, and sulfur metabolism pathways.

The substrate proteins of ClpP are various in bacteria, and the substrates of ClpP in phytopathogenic bacteria remained unknown. We then determined the substrates of ClpP in *R. solanacearum* using iTRAQ proteomics analysis. *R. solanacearum* wild-type strain GMI1000 and mutant Δ*clpP* were cultured in BG medium to the same concentration of logarithmic growth phase with two biological repeats. Total proteins of the wild-type strain and mutant were extracted, labeled, fractionated, identified, and quantitated. A total of 3,059 proteins were identified and quantitated. Setting fold change > 1.5 with *P* ≤ 0.05 as the threshold, 59 proteins were downregulated and 85 proteins were upregulated in Δ*clpP* compared with that in the wild-type strain (Figure 3A and Supplementary Table S3).

The differentially expressed proteins were then analyzed by KEGG pathway enrichment. As shown in Figure 3B, the bacterial chemotaxis, flagellar assembly, and two-component system were the most enriched pathways. A total of 33 proteins were predicated in the bacterial chemotaxis pathway in *R. solanacearum* GMI1000. Twenty of these 33 proteins, including CheA, CheB, CheR, CheW, CheY, and CheZ, were upregulated in mutant Δ*clpP* compared with that of the wild-type strain GMI1000 (Supplementary Table S4). Fifteen upregulated proteins were associated with flagellar assembly. Thirty-two of the 85 upregulated proteins were involved in bacterial chemotaxis or flagellar assembly (three proteins were shared by bacterial chemotaxis and flagellar assembly pathways, Figure 3C). Twenty of these 32 proteins were also mapped to the two-component system pathway. These results suggested that proteins related to chemotaxis and motility and their regulation were the major substrates degraded by ClpP in *R. solanacearum* under the tested condition.

Moreover, sulfur metabolism was significantly enriched in the differentially expressed proteins. Except for PLP-dependent transferase (WP_011004079.1, RS_RS20845), the other three sulfur metabolism proteins were upregulated in mutant Δ*clpP* (Figure 3C). These proteins included ATP-binding cassette domain-containing protein (WP_011001290.1, RS_RS06725), aliphatic sulfonate ABC transporter permease SsuC (WP_011001289.1, RS_RS06720), and TauD/TfdA family dioxygenase (WP_011000701.1, RS_RS03760). These results indicated that these sulfur metabolism proteins might be the substrates of ClpP in *R. solanacearum*.

### ClpP Substrates Sulfur Metabolism Proteins Were Upregulated by Methyl Gallate

The differentially expressed proteins between MG-treated *R. solanacearum* and untreated *R. solanacearum* were identified by iTRAQ proteomics analysis as well. A total of 114 and 138 proteins were upregulated and downregulated by the MG treatment in *R. solanacearum* wild-type strain GMI1000 (Supplementary Table S5). MG treatment resulted in 166 upregulated proteins and 142 downregulated proteins in mutant Δ*clpP* (Supplementary Table S6). Moreover, 12 differentially expressed proteins caused by MG treatment in the wild-type strain GMI1000 were mapped to sulfur metabolism, which is the most significantly enriched pathway of the differentially expressed proteins (Supplementary Figure S1). However, sulfur metabolism was not enriched in the differentially expressed proteins caused by MG treatment in mutant Δ*clpP* (Supplementary Figure S2).

**FIGURE 4:**
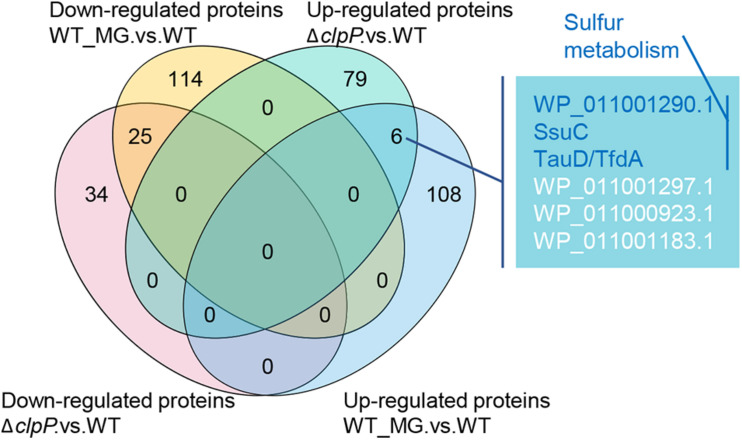
Differentially expressed proteins shared by Δ*clpP* versus wild-type (WT) and WT_MG versus WT.

The differentially expressed proteins caused by MG treatment and *clpP* gene mutation were then compared. As shown in Figure 4, MG-treated *R. solanacearum* and mutant Δ*clpP* shared six upregulated and 25 downregulated proteins. Three of the six shared upregulated proteins were the sulfur metabolism-associated proteins, namely, ATP-binding cassette domain-containing protein (WP_011001290.1), aliphatic sulfonate ABC transporter permease SsuC (WP_011001289.1), and TauD/TfdA family dioxygenase (WP_011000701.1). These results indicated that MG might inhibit ClpP activity and consequently disorder homeostasis of sulfur metabolism-associated proteins.

### Molecular Docking of ClpP and Methyl Gallate

**FIGURE 5:**
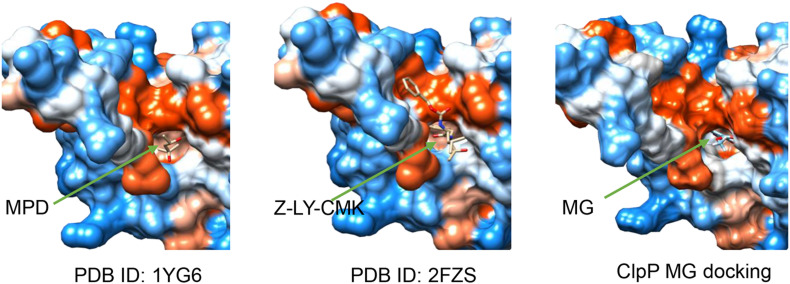
Structures of ClpP binding with (4S)-2-methylpentane-2,4-diol (MPD) (PDB ID: 1YG6), benzyloxycarbonyl-leucyltyrosine chloromethyl ketone (Z-LY-CMK) (PDB ID: 2FZS), and methyl gallate (MG) (the best docking pose). Ligands were indicated by green arrows.

The interaction between ClpP and MG was analyzed by molecular docking to validate whether ClpP was a target of MG. *R. solanacearum* ClpP shared 88% coverage and 67% identity with the ClpP of *E. coli*, the structure of which has been resolved (Bewley et al., 2006; Alexopoulos et al., 2012). ClpP oligomerizes as two stacked heptameric rings. Chain A of ClpP heptameric structure (PDB ID: 1YG6) was selected for molecular docking analysis using SwissDock (Grosdidier et al., 2011). MPD, a ligand that was added for ClpP crystal growing (Bewley et al., 2006), and ClpP inhibitors benzyloxycarbonyl-leucyltyrosine chloromethyl ketone (Z-LY-CMK) (Szyk and Maurizi, 2006) and 3,4-dichloroisocoumarin (3,4-DIC) (Sassetti et al., 2019) were used as control ligands docking with ClpP. The top-ranked docking poses were compared in Table 2. The best mode of MG docking pose provided the FullFitness of −1,121.26 kcal/mol and estimated ΔG of −6.81 kcal/mol, which are better than the score of 3,4-DIC. The best predicted binding mode between ClpP and MG was then visualized by UCFS Chimera (Pettersen et al., 2004). The structures of ClpP binding MPD (PDB ID: 1YG6) and Z-LY-CMK (PDB ID: 2FZS) were also compared. As shown in Figure 5, MPD, Z-LY-CMK, and MG were bound at the hydrophobic pocket of ClpP, which is the key active site. The molecular docking confirmed the possible interaction between ClpP and MG.

**TABLE 2:** Results of ClpP docking with certain ligands.

Ligands	Ligands structure	FullFitness (kcal/mol)	Estimated Δ G (kcal/mol)
MG	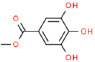	−1,121.26	−6.81
MPD	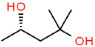	−1,137.62	−6.48
Z-LY-CMK	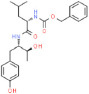	−1,143.03	−8.88
3,4-DIC	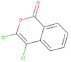	−1,091.61	−6.33

*MG, methyl gallate; MPD, (4S)-2-methylpentane-2,4-diol; Z-LY-CMK, benzyloxycarbonyl-leucyltyrosine chloromethyl ketone; 3,4-DIC, 3,4-dichloroisocoumarin.*

## Discussion

Apart from ClpP, the membrane protein RS_RS01640 was identified as the possible target of MG by Tn-seq. The transposon insertion of *RS_RS01640* resulted in an increase in relative fitness under the treatment. However, the *RS_RS01640*-deleted mutant did not show obvious resistance to MG. One possibility is that the growth attenuation caused by the deletion of *RS_RS01640* masked the resistance to MG, and a more sensitive method is needed to confirm the effect of RS_RS01640 on MG. Another possibility is that all transposon-inserted mutants were cultured en masse in a pool, and the relative abundance of a certain mutant might be affected by other mutants or by public goods, which makes the results from Tn-seq differ from the phenotypes of each individual (Thibault et al., 2019).

The Clp proteolytic complex is composed of ClpP associated with subunits of either of two Clp ATPases, ClpA or ClpX. As shown in Supplementary Figure S3A, ClpP subunits are organized in two stacked heptameric rings enclosing a central chamber containing the proteolytic sites. ClpX or ClpA binds both axial surfaces of the ClpP tetradecamer forming a barrel-like complex (Alexopoulos et al., 2012). In the absence of ClpX or ClpA, ClpP degrades only small peptides instead of specific substrates (Woo et al., 1989). *clpX* (*RS_RS08650*) lies adjacent to *clpP* in the genome of *R. solanacearum* GMI1000. We then deleted *clpX* in-frame and assayed the MG resistance of *clpX*-deleted mutant Δ*clpX*. However, *clpX* deletion neither alters the growth of *R. solanacearum* nor affects MG resistance (Supplementary Figure S3B). This indicates that ClpP but not ClpX is targeted by MG. *clpP* mutant of *S. aureus* was found sensitive to conditions generating misfolded proteins, whereas the absence of *clpX* improved survival (Frees et al., 2003). Clp ATPases ClpA and ClpX confer distinct substrates to the Clp protease complexes (Gottesman et al., 1998). Whether the proteolytic complex composed of ClpP and ClpA is a target of MG needs further research.

MG inhibits the growth of *R. solanacearum* and reduces the incidence of tomato bacterial wilt. *clpP* deletion increased the resistance of *R. solanacearum* to MG *in vitro*. The MG biocontrol efficacy on the tomato bacterial wilt caused by *R. solanacearum* wild-type strain and mutant Δ*clpP* was assayed. However, Δ*clpP* did not cause any bacterial wilt symptoms on the tomato by soil drenching infection. The role of ClpP in bacterial pathogenesis has been widely reported. ClpP is essential for the pathogenesis of *S. typhimurium*, *S. aureus*, *L. monocytogenes*, and *S. pneumonia* (Bhandari et al., 2018). However, the role of ClpP in phytopathogenic bacterial pathogenesis remains unknown and needs further study.

ClpP together with Lon proteases perform the majority of the bacterial proteolytic activities and may be responsible for about 80% of cellular proteolysis (Goldberg et al., 1994). *Caulobacter crescentus* undergoes a dimorphic life cycle comprising the obligate differentiation of a motile swarmer cell to a non-motile stalked cell. The differentiation of the swarmer cell into a stalked cell is accompanied by the degradation of the flagellar and chemotaxis apparatus *via* ClpP (Tsai and Alley, 2001; Potocka et al., 2002). In this study, the substrates of ClpP in *R. solanacearum* were identified by iTRAQ proteomics analysis. The proteomics study showed that 32 of the 85 possible substrate proteins, which were upregulated in Δ*clpP*, were involved in bacterial chemotaxis or flagellar assembly. *R. solanacearum* strains used for iTRAQ proteomics analysis were cultured in liquid BG medium shaking at 200 rpm. *R. solanacearum* cells were subjected to passive motion, and chemotaxis and flagella might be useless for bacterial survival under this growing condition. Similar to the cell differentiation in *C. crescentus*, the flagellar and chemotaxis apparatus of *R. solanacearum* may be degraded *via* ClpP to adapt to the given condition. We deduce that the substrates of the ClpP may vary according to the bacterial growing condition and growth phase. More conditions would be tested to obtain the full repertoire of ClpP substrates.

Through Tn-seq and gene deletion in-frame, this study found that the absence of ClpP caused the resistance to MG in *R. solanacearum*. Sulfur metabolism-associated proteins, ClpP substrates identified by iTRAQ, were upregulated by MG treatment. Moreover, molecular docking confirmed the possible interaction between ClpP and MG. Collectively, we infer that bacterial ClpP is a potential target for MG, and MG is a potential inhibitor of ClpP.

## Data Availability Statement

The datasets presented in this study can be found in online repositories. The names of the repository/repositories and accession number(s) can be found below: (Figshare: 10.6084/m9.figshare.12869930; ProteomeXchange Consortium: PXD021102).

## Author Contributions

DZ designed the study, performed the study, analyzed the data, and revised the manuscript. YX drafted the manuscript. GY, XW, and QL took part in the conception of this study and revised the manuscript. All the authors edited the manuscript and approved the final manuscript.

## Conflict of Interest

The authors declare that the research was conducted in the absence of any commercial or financial relationships that could be construed as a potential conflict of interest.
