# Supplementary material for: Bacterial ClpP Protease Is a Potential Target for Methyl Gallate
Source: Front Microbiol. 2021 Feb 4;11:598692. doi: 10.3389/fmicb.2020.598692 (PMC7890073; doi:10.3389/fmicb.2020.598692)
Supplement: Supplementary file 1 [file Data_Sheet_1.docx]

**Figure S1** KEGG enrichment of differentially expressed proteins of WT_MG versus WT. The numbers of differentially expressed proteins are indicated by the size of bubbles. *P* values of enrichment are indicated by the color of bubbles. The ratio represents the number of proteins identified in this pathway divided by the number of differentially expressed proteins mapped to this pathway. The top 20 over enriched KEGG pathways were shown.

**Figure S2** KEGG enrichment of differentially expressed proteins of Δ*clpP*_MG versus Δ*clpP*. The numbers of differentially expressed proteins are indicated by the size of bubbles. *P* values of enrichment are indicated by the color of bubbles. The ratio represents the number of proteins identified in this pathway divided by the number of differentially expressed proteins mapped to this pathway. The top 20 over enriched KEGG pathways were shown.

**Figure S3** Effect of clpX deletion on MG sensitivity. **A.** The reported Clp proteolytic complex that composed of ClpP and ClpX. **B.** Gradient diluted bacterial cultures were inoculated on BG agar medium added with or without 25μg/mL of MG. The growth of *R. solanacearum* strains was observed and photographed at certain times.
